# Supplementary material for: Explainable artificial intelligence for cough-related quality of life impairment prediction in asthmatic patients
Source: PLoS One. 2024 Mar 19;19(3):e0292980. doi: 10.1371/journal.pone.0292980 (PMC10950232; doi:10.1371/journal.pone.0292980)
Supplement: S1 Table — Structure of the questionnaire used to collect patients’ respiratory symptoms. The groups of items forming the features used in our analysis are defined as follows: items 1–3 (AsthmaRelated); 4–7 (PharynxLarynx); 8–14 (RhinoSinusitis); 15–18 (GastroEsoReflux). (PDF) [file pone.0292980.s001.pdf]

RESPIRATORY SYMPTOMS QUESTIONNAIRE

Date: ...../....../......

Surname and Name: ....

			How intense/annoying has the symptom been in the last month? (put an X on the row)
1	Shortness of breath	☐	None |______| Very much
2	Hisses / Whistles	☐	None |______| Very much
3	Chest tightness	☐	None |______| Very much
4	Sore/burning in the throat	☐	None |______| Very much
5	Hoarse voice, ease of losing voice	☐	None |______| Very much
6	Sense of closure in the throat	☐	None |______| Very much
7	Swallowing difficulty	☐	None |______| Very much
8	Snoring	☐	None |______| Very much
9	Stuffy nose	☐	None |______| Very much
10	Runny nose	☐	None |______| Very much
11	Phlegm between nose and throat	☐	None |______| Very much
12	Yellow phlegm from the nose	☐	None |______| Very much
13	Reduced sense of smell	☐	None |______| Very much
14	Pain in the face (forehead and cheekbones)	☐	None |______| Very much
15	Heartburn	☐	None |______| Very much
16	Regurgitation/ Acid in the throat	☐	None |______| Very much
17	Nausea	☐	None |______| Very much
18	Cough when lying down	☐	None |______| Very much
